# Racial and Ethnical Discrepancies and Similarities in the Epidemiology, Survival, and Neurological Outcomes After Acute Traumatic Spinal Cord Injury: A Retrospective Cohort Study Using Data from the NASCIS-1 Trial

**DOI:** 10.46292/sci23-00055S

**Published:** 2023-11-17

**Authors:** Julio C. Furlan

**Affiliations:** 1 Lyndhurst Centre, Toronto Rehabilitation Institute, University Health Network, Toronto, ON, Canada;; 2 KITE Research Institute, University Health Network, Toronto, ON, Canada;; 3 Department of Medicine, Division of Physical Medicine and Rehabilitation, University of Toronto, Toronto, ON, Canada;; 4 Rehabilitation Sciences Institute, University of Toronto, Toronto, ON, Canada; 5 Institute of Medical Science, University of Toronto, Toronto, ON, Canada;; 6 Institute of Health Policy, Management and Evaluation, University of Toronto, Toronto, ON, Canada

**Keywords:** ethnicity, race, neurological recovery, spinal cord injury, survival, trauma

## Abstract

**Background:**

Little is known about the impact of race/ethnicity on the clinical and neurological outcomes after acute traumatic spinal cord injury (tSCI).

**Objectives:**

This study examined the influence of race/ethnicity on the individuals’ survival and neurological recovery within the first year after tSCI.

**Methods:**

The 306 cases enrolled in the First National Acute Spinal Cord Injury Study (NASCIS-1) were grouped as African American individuals (*n* = 84), non-Hispanic White individuals (*n* = 159), and other races/ethnicities that included Hispanic individuals (*n* = 60) and Asian individuals (*n* = 3). Outcome measures included survival and neurological recovery within the first year after tSCI. Data analyses were adjusted for major potential confounders.

**Results:**

There were 39 females and 267 males with mean age of 31 years who mostly sustained cervical severe tSCI after motor vehicle accidents or falls. The three groups were comparable regarding sex distribution, level and severity of tSCI, level of consciousness at admission, and total received dose of methylprednisolone. African American individuals were significantly older than non-Hispanic White individuals (*p* = .0238). African American individuals and individuals of other races/ethnicities more often had a tSCI with open wound caused by missile and water-related accidents than non-Hispanic White individuals (*p* < .0001). Survival rates within the first year after tSCI were comparable among the three groups (*p* = .3191). Among the survivors, there were no significant differences among the three groups regarding motor and pinprick and light-touch sensory recovery (*p* > .0500).

**Conclusions:**

The results of this study suggest that, while there were few differences among the racial/ethnical groups regarding the epidemiology of tSCI, race/ethnicity did not influence survival rate or neurological recovery within the first year post-tSCI.

## Introduction

Traumatic spinal cord injury (tSCI) can result in substantial motor, sensory, and autonomic impairments to individuals. The worldwide incidence of tSCI varies from 6.2 to 174 per million inhabitants annually, and the global prevalence of tSCI ranges from 50 to 906 per million inhabitants.^1^ The epidemiology of tSCI is variable among the different geopolitical regions across the world with respect to age and sex distribution, level and severity of tSCI, and the distribution of racial and ethnic groups.^2^ Furthermore, there have been temporal changes in the epidemiology of tSCI due to, for instance, an aging population that has led to an increase in fall-related SCI in the geriatric population over the past decades across many jurisdictions.^3^-^5^ Moreover, the proportions of individuals from different racial and ethnic groups have altered in certain geopolitical regions. According to data from the National Spinal Cord Injury Database, the racial/ethnical distribution between 1973 and 1979 included 76.8% Caucasian individuals, 14.2% African American individuals, and 0.9% Asian individuals with acute tSCI, whereas there were 66.5% Caucasian individuals, 26.8% African American individuals, and 2.0% Asian individuals with acute tSCI between 2005 and 2010.^6^

There is a growing body of evidence in the literature that suggests that race/ethnicity has a significant impact on epidemiology and/or outcomes of other neurological diseases or injuries of the central nervous system. For instance, Amezcua et al.^7^ reported that “neuromyelitis optica spectrum disorder disproportionately affects Black individuals, and multiple sclerosis has seen a recent shift in select racial groups.” Robinson et al.^8^ documented that Black individuals are more susceptible to recurrent stroke, even though proper management of the traditional stroke risk factors could mitigate those racial/ethnical disparities in stroke recurrence. Brenner et al.^9^ found that the incidence of traumatic brain injury (TBI) in Black individuals was greater than expected based on their representation in the state of Pennsylvania in 2010, whereas the incidence of TBI in Asian individuals was lower than expected. Moreover, Warren et al.^10^ reported significant racial/ethnic discrepancies in functional outcomes during inpatient rehabilitation for TBI; for example, Black race/ethnicity was associated with statistically significant lower cognitive, motor, and total efficiency Functional Independence Measure (FIM) scores and worse functionality scores at discharge compared to non-Hispanic White race/ethnicity.

Given that little is known about the impact of race/ethnicity on the clinical and neurological outcomes of individuals following acute tSCI, a retrospective cohort study using a prospectively accrued data was undertaken to assess the influence of race and ethnicity on the individuals’ survival and neurological recovery within the first year after tSCI. This study particularly addressed a knowledge gap in the potential effects of race/ethnicity on clinically relevant outcomes after tSCI using high-quality data from the First National Acute Spinal Cord Injury Study (NASCIS-1) that were analyzed after controlling for major potential confounders. Furthermore, the participants were enrolled in NASCIS-1 between 1979 and 1981 when the standard of care could have been distinct from the current guidelines with respect to some aspects of prehospital, acute spine care, and rehabilitation practices. For instance, maintenance of spinal cord perfusion by properly managing mean arterial pressure (≥ 85 mmHg), preoperative evaluation using MRI, and early decompression of spinal cord have lately become the best practices in the management of patients with acute tSCI.^11^,^12^ In addition, the importance of equity, diversity, and inclusivity in healthcare was not appropriately recognized during the 70s and 80s as it is today, and thus this study represents a unique opportunity to indirectly evaluate the influence of race/ethnicity on the access to the best practices for management of individuals with acute tSCI. In a recent scoping review on traumatic injuries including tSCI, Moore et al.^13^ reported that there may be significant racial/ethnical disparities with regard to healthcare access in the acute care setting and rehabilitation setting. Moreover, they indicated that there is a paucity of research studies focused on traumatic injuries with respect to prevention and policy development to overcome racial/ethnical disparities.^13^

## Methods

The Institutional Ethics Board from the Yale University approved the use of the NASCIS-1 database to answer research questions other than the primary aims of the clinical trial.

This retrospective cohort study included all 306 individuals who were enrolled in the NASCIS-1 trial. This clinical trial was designed to compare the efficacy of a high dose of methylprednisolone (bolus of 1000 mg of MPSS and daily thereafter for 10 days) with a standard dose (bolus of 100 mg of MPSS and daily thereafter for 10 days) in the management of individuals with acute tSCI.^14^ All participants in the NASCIS trial were recruited to the study between February 1979 and November 1981 in one of the nine hospitals, including six specialized spinal cord centers, which were affiliated to Yale University (New Haven, CT), New York University (Bellevue, NY), Medical University of South Carolina (Charleston), Ohio State University (Columbus), University of Texas Medical Branch (Galveston), University of Miami (Miami, Florida), University of Puerto Rico (San Juan), Riverside Methodist Hospital (Columbus, OH), and Baylor College of Medicine (Houston, TX). Individuals with acute tSCI at the age of 14 years or older were considered eligible for the NASCIS-1 trial and were successively recruited to the NASCIS-1 trial. Individuals who were admitted in a participant center more than 48 hours after the injury and individuals with “severe comorbidities” or other specific reasons (“including a history of diabetes, severe vascular disease, concurrent infection, gastrointestinal tract bleeding and pregnancy”) were excluded. Complete inclusion and exclusion criteria are reported in detail elsewhere.^14^

### Baseline data

The NASCIS-1 database includes information on the individuals’ demographics (i.e., age at the time of tSCI, and sex), racial and ethnic groups (“Black individuals,” “White individuals,” and others including “Hispanic individuals” and “Asian individuals”), causes of tSCI (“fall,” “missile,” motor vehicle accident that includes “automobile accidents” and “motorcycle accidents,” “water related,” others that include “crush” and “unknown,” and “missing”), level of consciousness at admission (“normal” and “abnormal”), and type of wound (“open” and “closed”).

The degree of severity of tSCI was classified using Frankel grades.^15^ The level of tSCI was categorized as “tetraplegia/tetraparesis,” “paraplegia/paraparesis,” and “minor neurological deficits without specified level.”

### Outcome measures

Survival data were analyzed for the first year after tSCI. The degree of impairment was assessed using the NASCIS motor and sensory scores in all individuals at admission in the emergency department and at 1 year after tSCI. The NASCIS motor scores varied from 14 (all normal motor responses) to 84 (no contraction in any muscle).^14^ Pinprick and light touch sensory scores varied from 29 (all normal sensory responses) to 87 (all absent sensory responses) each one.^14^

Neurological recovery at 1 year after tSCI was determined by subtracting the baseline NASCIS score at admission from the follow-up NASCIS score at 1 year after injury, hence a negative neurological recovery score represents improved function whereas a positive score characterizes decreased function.

### Study groups and sensitivity analyses

Based on the information available in the NASCIS-1 database, the participants were grouped according to their race/ethnicity into (a) African American individuals (*n* = 84), (b) non-Hispanic White individuals (*n* = 159), and (c) others that included Hispanic individuals (*n* = 60) and Asian individuals (*n* = 3). A series of sensitivity analyses was performed imputing hypothetical data to replace missing data on the 1-year follow-up Frankel grades. In the “best case scenario” sensitivity analysis, all cases with missing data were assumed to have improved at least one degree in the Frankel score (including one or more degrees of improvement) from baseline to 1-year follow-up assessment. For the “worst case scenario” sensitivity analyses, all cases with missing data were theoretically considered as they have had no improvement or decline in the Frankel grade from the baseline to 1-year follow-up assessment.

### Data analysis

The three racial/ethnic groups were statistically compared with respect to their baseline data using analysis of variance (ANOVA) with Bonferroni post hoc test and Fisher exact test or chi-square test. Data on survival within the first year after tSCI was analysed using Fisher exact test and Kaplan-Meier curve with log-rank test.

Multiple regression analyses were used to evaluate the potential effects of the race/ethnicity on the neurological recovery (motor recovery, and pinprick and light-touch sensory recovery) at 1 year after tSCI. Those multiple regression analyses were adjusted for major potential confounders including the individuals’ sex, age at tSCI onset, level of SCI, cause of injury, type of wound, level of consciousness at admission, and total received dose of MPSS.^14,16-25^

Finally, multiple logistic analyses were used to evaluate whether improved neurological recovery (defined as at least one-grade improvement in the 1-year follow-up Frankel grading) was associated with age after adjusting for the same major potential confounders. Similar multiple logistic regression analyses were performed for the “worst case scenario” and “best case scenario” sensitivity analyses.

Significance was assumed at *p* < .05. All data analyses had a minimum power of 80%. Data analyses were performed using SAS 9.4 (SAS Institute Inc., Cary, NC).

## Results

The study included 306 individuals (39 females and 267 males) with a mean age of 31 years (age range, 14 to 81 years) who sustained paraplegia (36.27%), tetraplegia (35.29%), tetraparesis (11.76%), paraparesis (6.21%), or minimal neurological deficit (10.46%). Of the 306 individuals with acute tSCI enrolled in the NASCIS-1 trial, there were 84 African American individuals, 159 non-Hispanic White individuals, and 63 individuals of other race/ethnicity. The three groups were comparable regarding sex distribution, level and severity of tSCI, level of consciousness at admission, and total received dose of MPSS in the acute stage post-tSCI (**Table 1**). However, African American individuals were significantly older than non-Hispanic White individuals with tSCI (**Table 1**). African American individuals and individuals of other races/ethnicities more often had a tSCI caused by missile and water-related accidents that more frequently resulted in open wounds than non-Hispanic White individuals, who had a greater proportion of tSCIs caused by motor vehicle accidents and fall-related accidents (**Table 1**). There was a trend toward a greater proportion of older individuals (group of individuals with 65 years of age or older) in the African American group than the other two study groups, but this did not reach statistical significance (**Table 1**).

**Table 1.** Baseline data comparing racial/ethnic groups of individuals with acute tSCI who were enrolled into the NASCIS-1 trial

**Characteristics**	**African American individuals(*n* = 84)**	**Other races/ethnicities(*n* = 63)**	**Non-Hispanic White individuals(*n* = 159)**	***p* values**
Mean age ± SEM, years	33.89 ± 1.60*	33.24 ± 1.88	28.91 ± 1.06*	.0238
Age range, years	14 to 69	14 to 65	15 to 81	
Age-related group				
Younger individuals	77 (91.67%)	62 (98.41%)	154 (96.86%)	
Older individuals (65 years of age or older)	7 (8.33%)	1 (1.59%)	5 (3.14%)	.0813
Sex				
Females	7 (8.33%)	7 (11.11%)	25 (15.72%)	
Males	77 (91.67%)	56 (88.89%)	134 (84.28%)	.2358
Cause of tSCI				
Motor vehicle accident	21 (25.30%)	19 (28.57%)	79 (46.54%)	
Fall	19 (22.89%)	18 (30.16%)	29 (18.24%)	
Missile	28 (33.73%)	19 (30.16%)	11 (6.92%)	
Water related	2 (2.41%)	5 (7.94%)	35 (22.01%)	
Other causes	13 (15.66%)	2 (3.17%)	10 (6.29%)	<.0001
*Missing data*	*1*	*0*	*0*	
Level of tSCI				
Tetraplegia/tetraparesis	40 (47.62%)	23 (36.51%)	81 (50.94%)	
Paraplegia/paraparesis	38 (45.24%)	34 (53.97%)	58 (36.48%)	
Minimal neurological deficit without specified level	6 (7.14%)	6 (9.52%)	20 (12.58%)	.1317
Severity of tSCI				
Frankel grade A	54 (64.30%)	37 (58.73%)	75 (47.17%)	
Frankel grade B	10 (11.90%)	6 (9.52%)	20 (12.88%)	
Frankel grade C	10 (11.90%)	8 (12.70%)	27 (16.98%)	
Frankel grade D	10 (11.90%)	10 (15.87%)	35 (22.01%)	
Frankel grade E	0 (0%)	2 (3.17%)	2 (1.26%)	.2073
Type of wound				
Closed wound	54 (64.29%)	44 (69.84%)	146 (91.82%)	
Open wound	30 (35.71%)	19 (30.16%)	13 (8.18%)	<.0001
Level of consciousness at admission				
Normal	75 (89.29%)	59 (93.65%)	133 (83.65%)	
Altered	9 (10.71%)	4 (6.35%)	26 (16.35%)	.1060
Total received dose of MPSS				
Mean total dose ± SEM, mg	5542.32 ± 526.80	4990.51 ± 605.10	6203.75 ± 392.16	.2203
Total dose range, mg	800 to 11960	200 to 11860	100 to 15267	

*Note*: Continuous variables were analyzed using analysis of variance (ANOVA) with Bonferroni *post hoc* test (any significant difference was indicated) and categorical variables were analyzed using Fisher exact test. NASCIS-1 = First National Spinal Cord Injury Study; MPPS = methylprednisolone; SEM = standard error of mean; tSCI = traumatic spinal cord injury.

* *p* = .0090 based on the results of ANOVA with Bonferroni's correction.

### Survival analysis

**Figure 1.**
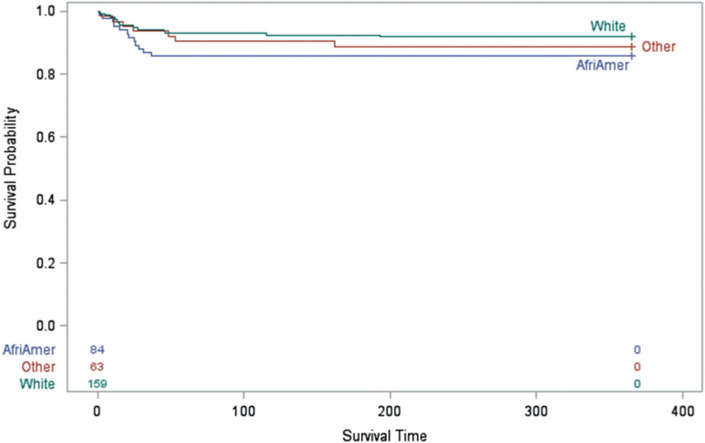
Survival analysis within the first year after acute traumatic spinal cord injury comparing African Americans individuals (AfriAmer), non-Hispanic White individuals (White), and individuals of other races/ethnicities (other), using Kaplan-Meier analysis with log-rank test (*p* = .3191).

The 1-year mortality rates post-tSCI among African American individuals (14.29%), non-Hispanic White individuals (8.18%), and individuals of other races/ethnicities (11.11%) were not statistically significant different (*p* = .3284) in a univariate analysis with a minimum power of 80%. Survival analysis using Kaplan-Meier curve and log-rank test showed that there were no statistically significant differences regarding the survival rates within the first year post-tSCI among the racial/ethnic groups (*p* = .3191; **Figure 1**).

### Neurological recovery

**Figure 2.**
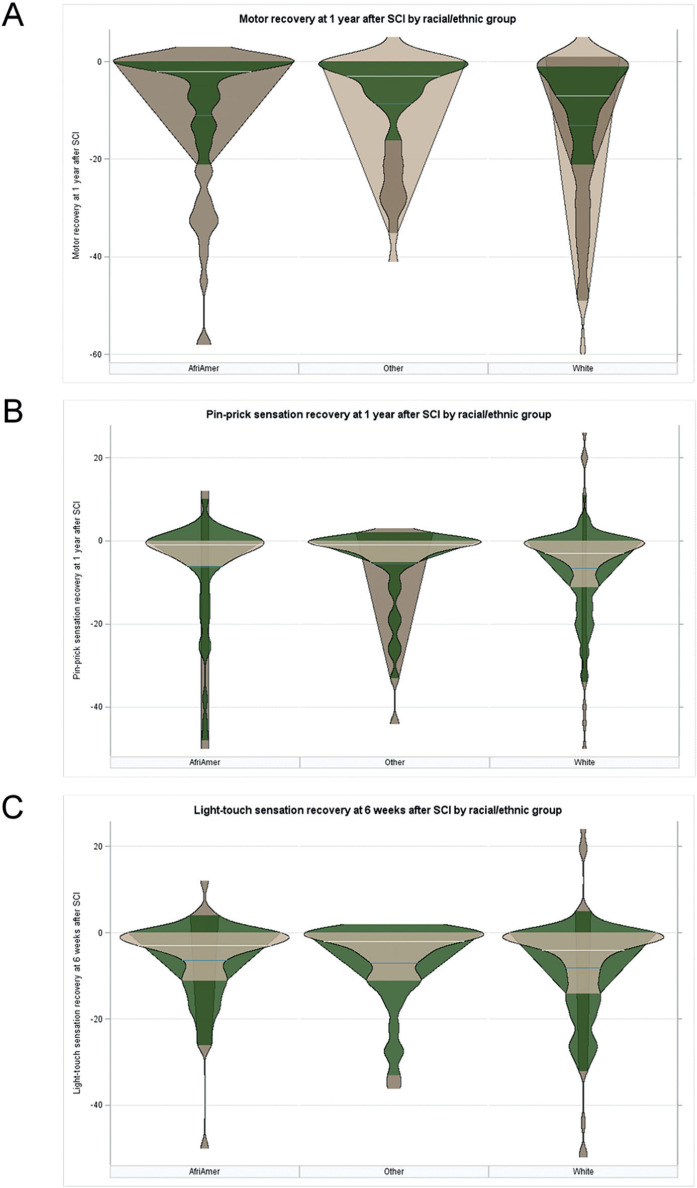
Results of univariate analyses comparing African American individuals (AfriAmer), non-Hispanic White individuals (White), and individuals of other races/ethnicities (other) with regards to their neurological recovery at 1 year after acute traumatic spinal cord injury as assessed by (**A**) the NASCIS motor score (*p* = .2231), (**B**) the NASCIS pinprick sensory score (*p* = .8606), and (**C**) the NASCIS light-touch sensory score (*p* = .5965).

Among survivors, there were no significant differences among the racial/ethnical groups regarding their motor recovery at 1 year post-tSCI (*p* = .2231; **Figure 2A**), pinprick sensory recovery at 1 year post-tSCI (*p* = .8606; **Figure 2B**), or light-touch sensory recovery at 1 year post-tSCI (*p* = .5965; **Figure 2C**).

Using multiple regression analysis adjusted for major potential confounders, race/ethnicity did not significantly influence motor recovery (model 1), pinprick sensory recovery (model 2), and light-touch sensory recovery (model 3) at 1 year post-tSCI (**Table 2**).

**Table 2:** Results of the multiple regression analysis on the effects of race/ethnicity on the motor (model 1), pinprick sensory recovery (model 2), and light-touch sensory recovery (model 3) at 1 year after acute tSCI

**Model 1: *Motor recovery at 1 year as assessed using NASCIS motor score***						
***R*^2^ = 0.1735; *F* value = 3.36; *p* = .0001**						
**Parameter**	**Estimate**	**SE**	***t***	***p* > [*t*]**	**95% confidence limits**	
Intercept	-7.5964	10.8246	-0.7000	0.4836	-28.9364	13.7436
Race: African American	0.1972	2.4227	0.0800	0.9352	-4.5789	4.9734
Race: other races/ethnicities	1.0776	2.7308	0.3900	0.6935	-4.3059	6.4612
Race: non-Hispanic White	*Reference*	.	.	.	.	.
Age, years	-0.2855	0.0817	-3.4900	0.0006	-0.4466	-0.1244
Sex: Female	-3.5274	2.8977	-1.2200	0.2249	-9.2401	2.1853
Sex: Male	*Reference*	.	.	.	.	.
Cause: Fall	6.6629	3.6979	1.8000	0.0730	-0.6273	13.9531
Cause: MVA	2.0316	3.0861	0.6600	0.5111	-4.0524	8.1157
Cause: Missile	9.5971	10.9543	0.8800	0.3820	-11.9987	31.1928
Cause: Other	2.1389	4.4231	0.4800	0.6292	-6.5809	10.8586
Cause: Water	*Reference*	.	.	.	.	.
Level: Minor injury	9.4474	3.3427	2.8300	0.0052	2.8575	16.0373
Level: Para	1.8680	2.2255	0.8400	0.4022	-2.5194	6.2554
Level: Tetra	0.0000	.	.	.	.	.
LOC: Abnormal	-5.5058	3.0343	-1.8100	0.0710	-11.4878	0.4762
LOC: Normal	*Reference*	.	.	.	.	.
Wound: Closed	-0.2422	10.2520	-0.0200	0.9812	-20.4533	19.9689
Wound: Open	*Reference*	.	.	.	.	.
Total MPSS dose	-0.0001	0.0002	-0.4300	0.6657	-0.0005	0.0003
**Model 2: *Pinprick sensory recovery at 1 year as assessed using NASCIS sensory score***						
*R*^2^ = 0.1223; *F* value = 2.23; *p* = .0001						
**Parameter**	**Estimate**	**SE**	***t***	***p* > [t]**	**95% confidence limits**	
Intercept	-17.3521	8.4738	-2.0500	0.0418	-34.0578	-0.6465
Race: African American	-0.9854	1.8965	-0.5200	0.6039	-4.7243	2.7536
Race: other races/ethnicities	-1.5432	2.1377	-0.7200	0.4712	-5.7576	2.6712
Race: non-Hispanic White	*Reference*	.	.	.	.	.
Age (in years)	-0.1129	0.0640	-1.7600	0.0792	-0.2390	0.0133
Sex: Female	0.3702	2.2684	0.1600	0.8705	-4.1018	4.8423
Sex: Male	*Reference*	.	.	.	.	.
Cause: Fall	5.6755	2.8948	1.9600	0.0513	-0.0315	11.3825
Cause: MVA	5.2186	2.4159	2.1600	0.0319	0.4558	9.9814
Cause: Missile	16.5936	8.5754	1.9400	0.0543	-0.3123	33.4995
Cause: Other	7.3828	3.4625	2.1300	0.0342	0.5567	14.2089
Cause: Water	*Reference*	.	.	.	.	.
Level: Minor injury	5.1870	2.6168	1.9800	0.0488	0.0283	10.3458
Level: Para	4.1536	1.7422	2.3800	0.0180	0.7190	7.5882
Level: Tetra	*Reference*	.	.	.	.	.
LOC: Abnormal	-3.3054	2.3754	-1.3900	0.1656	-7.9883	1.3775
LOC: Normal	*Reference*	.	.	.	.	.
Wound: Closed	9.3372	8.0256	1.1600	0.2460	-6.4847	25.1591
Wound: Open	*Reference*	.	.	.	.	.
Total MPSS dose	-0.0002	0.0002	-1.2800	0.2023	-0.0005	0.0001
**Model 3: *Light-touch sensory recovery at 1 year as assessed using NASCIS sensory score***						
*R*^2^ = 0.0997; *F* value = 1.76; *p* = .0521						
**Parameter**	**Estimate**	**SE**	***t***	***p* > [t]**	**95% confidence limits**	
Intercept	-16.6124	8.2071	-2.0200	0.0442	-32.7931	-0.4316
Race: African American	1.7023	1.8424	0.9200	0.3566	-1.9300	5.3346
Race: other races/ethnicities	-0.4984	2.0763	-0.2400	0.8105	-4.5919	3.5951
Race: non-Hispanic White	*Reference*	.	.	.	.	.
Age (in years)	-0.0874	0.0622	-1.4100	0.1614	-0.2100	0.0352
Sex: Female	0.8987	2.2014	0.4100	0.6835	-3.4414	5.2388
Sex: Male	*Reference*	.	.	.	.	.
Cause: Fall	2.8156	2.8212	1.0000	0.3195	-2.7466	8.3777
Cause: MVA	2.2439	2.3419	0.9600	0.3391	-2.3733	6.8610
Cause: Missile	11.1585	8.3094	1.3400	0.1808	-5.2240	27.5409
Cause: Other	1.1066	3.3529	0.3300	0.7417	-5.5039	7.7171
Cause: Water	*Reference*	.	.	.	.	.
Level: Minor injury	6.8951	2.5864	2.6700	0.0083	1.7960	11.9942
Level: Para	3.8575	1.6909	2.2800	0.0235	0.5239	7.1912
Level: Tetra	*Reference*	.	.	.	.	.
LOC: Abnormal	-3.5884	2.3320	-1.5400	0.1254	-8.1860	1.0093
LOC: Normal	*Reference*	.	.	.	.	.
Wound: Closed	8.1496	7.7718	1.0500	0.2956	-7.1729	23.4720
Wound: Open	*Reference*	.	.	.	.	.
Total MPSS dose	-0.0001	0.0001	-0.6700	0.5043	-0.0004	0.0002

*Note*: Models were adjusted for major potential confounders (i.e., individuals’ sex, age at the time of injury, level of tSCI, cause of injury, type of wound, LOC at admission, and total received MPSS dose). tSCI = traumatic spinal cord injury; LOC = level of consciousness; MPSS = methylprednisolone; MVA = motor vehicle accident; NASCIS = National Spinal Cord Injury Study; Para = paraplegia or paraparesis; Tetra = tetraplegia or tetraparesis.

Finally, improved neurological recovery defined as at least one-grade enhancement in the Frankel grading from the baseline to the 1-year follow-up assessment was not adversely affected by race/ethnicity in a multiple logistic regression analysis adjusted for major potential confounders (**Table 3**). Similar results were obtained in the “best case scenario” sensitivity analysis when missing data on the 1-year follow-up Frankel grading was hypothetically assumed to be improved by at least one degree from the baseline assessment (**Table 3**). The results of the “worst case scenario” analysis indicated that race/ethnicity was not significantly associated with neurological recovery in most paired comparisons, if hypothetically there was no improvement of at least one-grade in the 1-year follow-up Frankel grading in all cases with missing follow-up assessments (**Table 3**). However, the results of the “worst case scenario” analysis suggested that the level of tSCI would have a significant impact on neurological recovery when comparing individuals with minor neurological deficits and individuals with tetraplegia (*p* = .0167; **Table 3**).

**Table 3:** Results of the multiple logistic analysis on the effects of race/ethnicity on the neurological improvement defined as at least one-grade enhancement in the Frankel grading from the initial to the 1-year follow-up assessment after acute tSCI

**Model 1: *Improved neurological recovery***				
**Model fit statistics: -2 log L = 244.3430 Likelihood ratio (chi-square): 18.3003; p = .1464 c statistics: 0.6710**				
**Covariates**	**Odds ratio**	**95% CI**		***p***
Race: African American vs. non-Hispanic White	1.2340	0.5510	2.7670	.8784
Race: Other races/ethnicities vs. non-Hispanic White	1.7230	0.6930	4.2820	.3228
Age	0.9920	0.9650	1.0200	.5873
Sex: Female vs. Male	0.6470	0.2580	1.6200	.3525
Cause: Fall vs. Water	2.0940	0.6680	6.5640	.9680
Cause: MVA vs. Water	1.2290	0.4830	3.1260	.2160
Cause: Missile vs. Water	5.0920	0.2080	124.4490	.4765
Cause: Other vs. Water	3.3640	0.6070	18.6590	.4649
Level: Minor vs. Tetra	0.2980	0.1000	0.8860	.0592
Level: Para vs. Tetra	0.6550	0.3170	1.3570	.6401
LOC: Abnormal vs. Normal	1.0380	0.3880	2.7780	.9407
Wound: Closed vs. Open	1.3610	0.0720	25.7470	.8372
Total MPSS dose, mg	1.0000	1.0000	1.0000	.5827
**Model 2: Improved neurological recovery *(“best case scenario” sensitivity)***				
**Model fit statistics: -2 log L = 294.9310 Likelihood ratio (chi-square): 20.7049; p = .0790 c statistics: 0.6640**				
**Covariates**	**Odds ratio**	**95% CI**	***p* value**
Race: African American vs. non-Hispanic White	0.9880	0.4770	2.0490	.5281
Race: Other races/ethnicities vs. non-Hispanic White	1.5250	0.6890	3.3730	.2597
Age	0.9680	0.9470	0.9900	.0043
Sex: Female vs. Male	0.6590	0.2930	1.4840	.3141
Cause: Fall vs. Water	1.5220	0.5370	4.3150	.5877
Cause: MVA vs. Water	1.2870	0.5330	3.1050	.3310
Cause: Missile vs. Water	5.3690	0.3270	88.2550	.3265
Cause: Other vs. Water	2.2740	0.5190	9.9640	.7130
Level: Minor vs. Tetra	0.5130	0.1810	1.4570	.2340
Level: Para vs. Tetra	0.8950	0.4720	1.6990	.5394
LOC: Abnormal vs. Normal	0.8330	0.3540	1.9580	.6750
Wound: Closed vs. Open	1.8760	0.1420	24.8130	.6329
Total MPSS dose, mg	1.0000	1.0000	1.0000	.7096
**Model 3: Improved neurological recovery *(“worst case scenario” sensitivity)***				
**Model fit statistics: -2 log L = 265.5800 Likelihood ratio (chi-square): 26.0369; p = .0168 c statistics: 0.6870**				
Covariates	Odds ratio	95% CI		*p* value
Race: African American vs. non-Hispanic White	1.2500	0.5650	2.7620	.8771
Race: Other races/ethnicities vs. non-Hispanic White	1.7640	0.7310	4.2550	.2874
Age	1.0110	0.9870	1.0350	.3749
Sex: Female vs. Male	0.7540	0.3240	1.7530	.5117
Cause: Fall vs. Water	2.2800	0.7630	6.8080	.8617
Cause: MVA vs. Water	1.2580	0.5080	3.1170	.2220
Cause: Missile vs. Water	3.8920	0.2110	71.6870	.5789
Cause: Other vs. Water	3.7720	0.6840	20.8070	.3263
Level: Minor vs. Tetra	0.2140	0.0720	0.6370	.0167
Level: Para vs. Tetra	0.5850	0.2930	1.1700	.5360
LOC: Abnormal vs. Normal	1.0480	0.4190	2.6250	.9201
Wound: Closed vs. Open	1.0240	0.0710	14.7830	.9858
Total MPSS dose, mg	1.0000	1.0000	1.0000	.6365

*Note*: Models were adjusted for major potential confounders (i.e. individuals’ sex, age at the time of injury, level of tSCI, cause of injury, type of wound, LOC at admission, and total received MPSS dose). LOC = level of consciousness; MPSS = methylprednisolone; MVA = motor vehicle accident; Para = paraplegia or paraparesis; Tetra = tetraplegia or tetraparesis; tSCI = traumatic spinal cord injury.

## Discussion

Despite a few differences among the racial/ethnical groups regarding their baseline data, the results of this study indicated that survival rates within the first year after acute tSCI were comparable among them. Among the survivors, the racial/ethnical groups were statistically comparable regarding individuals’ motor recovery, pinprick sensory recovery, and light-touch sensory recovery at 1 year post-tSCI, when using univariate analysis or multiple regression analysis adjusted for major potential confounders. Using a multiple logistic regression analysis adjusted for major potential confounders, race/ethnicity was not significantly associated with at least one-grade neurological improvement using Frankel grading from the baseline to the 1-year follow-up assessment. The robustness of those results was confirmed in the “worst case scenario” and “best case scenario” sensitivity analyses. While the “worst case scenario” sensitivity analysis had missing data replaced as if there was no improvement or decline in the 1-year follow-up Frankel grading, the “best case scenario” sensitivity analysis had missing data replaced as all those cases had an improvement of at least one-grade in the 1-year follow-up Frankel grading in all cases.

### Racial/ethnical discrepancies in the demographics and epidemiology of acute tSCI

The results of this study indicated a few differences among the racial/ethnical groups regarding their demographics and etiology and type of tSCI. More specifically, African American individuals were significantly older than non-Hispanic White individuals with tSCI, and there was a trend toward a greater proportion of older individuals in the African American group than the other two study groups, but it did not reach statistical significance. African American individuals and individuals of other races/ethnicities more frequently had a tSCI caused by missile and water-related accidents, which more frequently resulted in open wounds, than non-Hispanic White individuals, who had a greater proportion of tSCI caused by motor vehicle accidents and fall-related accidents.

Prior studies from the current literature reported conflicting results concerning the influence of race/ethnicity on the demographics and epidemiology of tSCI. Arango-Lasprilla et al.^26^ retrospectively analyzed data of 1528 individuals with tSCI, and they found significant differences among African American individuals, non-Hispanic White individuals, and Hispanic individuals regarding their age, sex distribution, and the cause of injury (i.e., violent versus nonviolent trauma). Analyzing data from 11,090 individuals with tSCI, Arango-Lasprilla et al.^27^ documented that African American individuals, non-Hispanic White individuals, and Hispanic individuals were significantly different regarding their age at the tSCI onset, sex distribution, level and severity tSCI, and cause of injury. In another retrospective study, Lad et al.^28^ studied 18,671 of cases of tSCI, which were grouped as African American individuals, Caucasian individuals, Hispanic individuals, Asian individuals, and Native American individuals, and they reported significant differences among those groups regarding their age, sex distribution, type of tSCI (penetrating vs. blunt vs. other types), severity of injuries, and the frequency of concomitant traumatic brain injury. In a cross-sectional study including 1063 individuals with tSCI, Cao et al.^29^ found significant discrepancies regarding age, sex distribution, level and severity of tSCI, and cause of injury among Hispanic individuals, African American individuals, non-Hispanic White individuals, and others (including American Indian, Asian, and Native Hawaiian individuals).

Overall, discrepancies in the demographics and epidemiology of tSCI are common findings in many prior studies and in this retrospective cohort study. Nevertheless, there is no consistency in the results from different studies that would suggest a particular tendency or bias. The reasons for those discrepancies remain incompletely understood, even though variations in the population racial/ethnical distribution and socioeconomic factors (such as income, employment, housing, community safety, and education) could explain some of those racial/ethnical differences in the demographics and epidemiology of tSCI.^13^

### Impact of race/ethnicity on outcomes following acute tSCI

The results of this study suggest that race/ethnicity had no impact on the 1-year survival rates post-tSCI, which is consistent with the results from two prior studies in the literature. Lad et al.^28^ studied inhospital survival rates amongst 18,671 individuals with tSCI who were grouped into White individuals (66.88%), African American individuals (16.91%), Hispanic individuals (9.93%), Asian individuals (1.75%), American Indian individuals (0.60%), Native Hawaiian individuals (0.09%), and other races/ethnicities (3.84%). The authors found no significant differences in survival among the racial/ethnical groups after controlling for the individual and injury characteristics. Dru et al.^30^ analyzed the in-hospital survival rates of 21,985 individuals with cervical tSCI with fracture, and they documented no significant differences regarding the in-hospital survival rates among the following racial/ethnic groups: Caucasian individuals, African American individuals, Hispanic individuals, Asian individuals, Native American individuals, and others.

Among the survivors in this study, the three racial/ethnical groups were comparable regarding their motor recovery, pinprick sensory recovery, and light-touch sensory recovery at 1 year post-tSCI, when using univariate analysis or using multiple regression analysis or multiple logistic regression analyses adjusted for major potential confounders. Similarly, Meade et al. 31 found no significant differences between a group of 314 African American individuals and a group of 314 White individuals in regard to their American Spinal Cord Injury (ASIA) motor scores at admission to the acute care and rehabilitation facilities and at discharge from the rehabilitation facility, as well as their ASIA motor change from rehabilitation admission to discharge. Furthermore, both racial/ethnical groups showed similar FIM motor scores at admission and discharge and the same FIM motor change from rehabilitation admission to discharge.^31^ Notably, the group of African American individuals was matched case for case based on age group, level and completeness of injury, and sponsor of care to the group of White individuals.^31^

Although there is a paucity of studies on the influence of race/ethnicity on the neurological recovery post-tSCI in the literature, several prior studies examined the effects of race/ethnicity on the degree of disability and handicap following tSCI. Putzke et al.^32^ compared a group of 187 African American individuals to a group of 187 White individuals, where individuals included in each group were matched case for case based on their age, level of education, gender, primary occupational status at the time of injury, level and severity of tSCI, cause of injury, and primary insurance sponsorship. The authors reported that both racial/ethnic groups reached similar functional recovery by the time of discharge from the rehabilitation facility, as assessed using FIM motor subscore and the FIM efficiency score.^32^ Fyffe et al.^33^ documented that African American individuals, non-Hispanic White individuals, and Hispanic individuals with motor complete tSCI had similar FIM self-care and mobility change scores from rehabilitation admission to rehabilitation discharge and at 1 year post-tSCI. However, African American individuals with motor complete tetraplegia or paraplegia had significantly poorer improvements in FIM self-care and mobility scores than non-Hispanic White individuals and Hispanics at discharge from the rehabilitation facility.^33^ The authors suggested that cultural factors or reduced access to economic and supportive resources during inpatient rehabilitation could explain those discrepancies in functional outcomes among the racial/ethnical groups.^33^

Although race/ethnicity does not appear to adversely affect the survival or neurological and functional recovery of individuals with acute tSCI, prior studies reported that race/ethnicity may have an impact on other post-tSCI outcomes, such as quality of life and satisfaction, employment outcomes, and secondary medical conditions.^34^,^35^ Racial/ethnic differences in various post-tSCI outcomes underscore the areas of medical, social, and economic science that can benefit from more equitable research. With more equitable research, efforts can be directed toward ensuring equitable access to the best practices of rehabilitation and participation for all individuals after tSCI.

## Study Limitations

This retrospective study analyzed high-quality data from the NASCIS-1 trial. Nonetheless, there are limitations that should be taken into account when interpreting and applying the results of this study. Firstly, the retrospective nature of the study did not allow inclusion of other potential confounders that could have an impact on the results. For instance, preexisting medical co-morbidities could affect not only survival but also neurological recovery after acute tSCI.^36^ Secondly, some potential discrepancies have been reported related to race/ethnicity regarding the quality and completeness of the data, which could also influence the results of the studies. For example, Chen et al.^37^ documented that Spinal Cord Injury Model Systems centers with higher rates of loss of follow-up assessments seemed to have a greater proportion of Hispanic individuals and African American individuals than White individuals. Thirdly, the participants were recruited for the NASCIS-1 trial between 1979 and 1981 when the standard of care was somewhat different from the current guidelines in terms of the prehospital, acute spine care, and rehabilitation practices. For example, maintenance of spinal cord perfusion by appropriately managing mean arterial pressure (≥ 85 mm Hg), preoperative MRI, and early decompression of spinal cord have latterly become the best practices in the management of patients with acute tSCI.^11^,^12^ Despite the differences in the management of individuals with acute tSCI, this study represented an unique opportunity to evaluate the influence of race/ethnicity on outcomes of patients after acute tSCI who were managed four decades ago under a standard of care different from the current practice guidelines. Over the past four decades, there have been changes in the demographics of tSCI with respect to age and race/ethnicity distribution. The importance of equity, diversity, and inclusivity in the acute care and rehabilitation of individuals with tSCI has also been increasingly recognized. Despite the epidemiological and social changes that have gradually emerged since the 70s, the results of this retrospective cohort study reinforce the notion that race/ethnicity is not a key biological determinant for survival and neurological recovery after tSCI.

## Conclusion

The results of this retrospective cohort study suggest that there might be some differences in the demographics and epidemiology of tSCI among African American individuals, non-Hispanic White individuals, and individuals of other racial/ethnical background. Nevertheless, race/ethnicity did not adversely affect the survival rate within the first year following acute tSCI. Moreover, the results of this study suggest that race/ethnicity did not influence the 1-year neurological recovery of individuals with acute tSCI. Altogether, those results reinforce the notion that race/ethnicity is not a key determinant for clinical and neurological outcomes after tSCI. This does not undermine the fact that there may be significant racial/ethnical disparities with regard to healthcare access in the acute care setting and rehabilitation setting as highlighted by Moore et al.^13^ in a recent scoping review on traumatic injuries including tSCI. Notably, there is also a paucity of research studies focused on traumatic injuries with respect to prevention and policy development to overcome racial/ethnical disparities.^13^ Those are important knowledge gaps that urge further investigations to further promote health equity in the field of tSCI.
